# Targeting GRP75 with a Chlorpromazine Derivative Inhibits Endometrial Cancer Progression Through GRP75–IP3R‐Ca^2+^‐AMPK Axis

**DOI:** 10.1002/advs.202304203

**Published:** 2024-02-11

**Authors:** Qi Wang, Lijuan Li, Xiaoyan Gao, Chunxue Zhang, Chen Xu, Lingyi Song, Jian Li, Xiao Sun, Fei Mao, Yudong Wang

**Affiliations:** ^1^ Department of Gynecologic Oncology the International Peace Maternity and Child Health Hospital School of Medicine Shanghai Municipal Key Clinical Specialty Female Tumor Reproductive Specialty Shanghai Key Laboratory of Embryo Original Disease Shanghai Jiao Tong University Shanghai 200025 China; ^2^ State Key Laboratory of Bioreactor Engineering Shanghai Frontiers Science Center of Optogenetic Techniques for Cell Metabolism Frontiers Science Center for Materiobiology and Dynamic Chemistry Shanghai Key Laboratory of New Drug Design School of Pharmacy East China University of Science and Technology Shanghai 200237 China

**Keywords:** chlorpromazine, endometrial cancer, GRP75, MAM

## Abstract

Tumors often overexpress glucose‐regulated proteins, and agents that interfere with the production or activity of these proteins may represent novel cancer treatments. The chlorpromazine derivative JX57 exhibits promising effects against endometrial cancer with minimal extrapyramidal side effects; however, its mechanisms of action are currently unknown. Here, glucose‐regulated protein 75 kD (GRP75) is identified as a direct target of JX57 using activity‐based protein profiling and loss‐of‐function experiments. The findings show that GRP75 is necessary for the biological activity of JX57, as JX57 exhibits moderate anticancer properties in GRP75‐deficient cancer cells, both in vitro and in vivo. High GRP75 expression is correlated with poor differentiation and poor survival in patients with endometrial cancer, whereas the knockdown of GRP75 can significantly suppress tumor growth. Mechanistically, the direct binding of JX57 to GRP75 impairs the structure of the mitochondria‐associated endoplasmic reticulum membrane and disrupts the endoplasmic reticulum–mitochondrial calcium homeostasis, resulting in a mitochondrial energy crisis and AMP‐activated protein kinase activation. Taken together, these findings highlight GRP75 as a potential prognostic biomarker and direct therapeutic target in endometrial cancer and suggest that the chlorpromazine derivative JX57 can potentially be a new therapeutic option for endometrial cancer.

## Graphical abstract

Glucose‐regulated protein 75 kD (GRP75) is a critical tether protein facilitating mitochondria‐associated endoplasmic reticulum membrane (MAM) formation through the IP3R–GRP75 complex and appears to be a promising therapeutic target in endometrial cancer. The present study shows that a chlorpromazine derivative, JX57, can directly exert its effect through the local GRP75 complex in endometrial cancer.

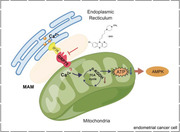

## 1 Introduction

Over the past three decades, endometrial cancer (EC) rates have steadily increased worldwide, especially in developed countries, where late childbearing is common.^[^ ^1^, ^2^ ^]^ As a result, women of reproductive age are increasingly affected in these regions.^[^ ^3^ ^]^ Although typical treatment for EC includes hysterectomy, the prevalence of this disease among reproductive‐age women has led to an interest in treatments that conserve reproductive function.^[^ ^4^ ^]^ The first‐line drug used to treat EC while conserving reproductive treatment is progesterone, but the response rates have not been high, and prolonged use may lead to drug resistance. Therefore, alternative therapeutic agents are urgently needed.

**Figure 1:**
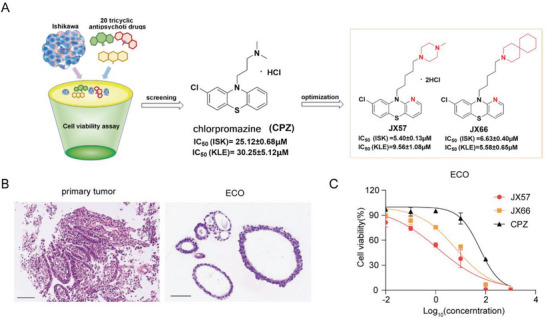
CPZ derivatives JX57 and JX66 exhibit anticancer properties in an endometrial cancer organoid (ECO) model. A) Schematic diagram showing the drug repurposing screening and structure modification strategy used to obtain the preferred compounds JX57 and JX66. B) Histological images showing the organization structure in primary tumor and ECO models. Scale bar: 100 µm. C) IC_50_ anti‐proliferation regression fitting curves for CPZ, JX57, and JX66 (concentration units = µm) generated using the ECO model; *n* = 3. Data are represented as mean ± SD.

Interestingly, antitumor properties have been identified in many established drugs.^[^ ^5^ ^]^ Through drug repurposing, previously approved or investigated drugs can be used in new ways that go beyond their original applications.^[^ ^6^ ^]^ In particular, the utilization of antipsychotics for EC has been an area of ongoing research, but the targets of these investigations remain unclear.^[^ ^7^ ^]^ Preliminary screening of 20 tricyclic antipsychotic drugs for an anti‐EC phenotype against 1400 approved drugs in our library revealed that the phenothiazine medicine chlorpromazine (CPZ), which has been widely used as an antipsychotic drug, also has strong anti‐EC activity. Through traditional drug structure optimization, the CPZ derivatives JX57 and JX66 were developed and found to have stronger anti‐EC activities than CPZ in vitro and in vivo (**Figure** **1**A), with minor extrapyramidal side effects.^[^ ^8^ ^]^ However, the molecular targets of JX57 and JX66 against EC remain unknown.

HSPA9 (GRP75/Mortalin) is a member of the heat shock 70 kDa protein family. Unlike most HSPs, GRP75 is found mainly in the endoplasmic reticulum (ER) and mitochondria, which are key organelles regulating protein quality control and metabolic homeostasis.^[^ ^9^ ^]^ Different subcellular localizations of GRP75 determine its functions in different cellular processes, including mitochondrial import, energy production, and cell cycle regulation.^[^ ^10^ ^]^ Disrupting GRP75 and P53 interactions is the main anticancer mechanism for GRP75 inhibitors.^[^ ^11^, ^12^, ^13^ ^]^ Sequencing results and published research have indicated that GRP75 is also found in the mitochondria‐associated endoplasmic reticulum membrane (MAM), but its complete local function has not been elucidated.^[^ ^14^, ^15^ ^]^ In the MAM, GRP75 serves as a tethering protein between the ER and mitochondria and regulates Ca^2+^ homeostasis.^[^ ^16^, ^17^ ^]^ ER–mitochondrial coupling is a key determinant of cell fate during cellular stress,^[^ ^18^, ^19^ ^]^ and ER–mitochondrial Ca^2+^ flux control regulates cell survival and mitochondrial function.^[^ ^16^, ^20^, ^21^ ^]^ The transport of Ca^2+^ from the ER to the mitochondria is mediated by the IP3R–GRP75 complex in the MAM and is critical for maintaining cellular bioenergy. These findings clearly show that GRP75 and its associated proteins are crucial for MAM function, and increasing evidence has suggested that GRP75 plays an important role in the occurrence and progression of cancer.^[^ ^22^, ^23^, ^24^, ^25^ ^]^ However, the role of GRP75 in EC and the potential underlying mechanisms remain unknown.

In this study, loss‐of‐function experiments and a series of functional assays were conducted to examine the anticancer properties of JX57 and the role of GRP75 in EC progression. Furthermore, the pharmacological mechanism, i.e., whether JX57 triggers a bioenergetic crisis to activate the AMP‐activated protein kinase AMPK pathway by inhibiting the GRP75–IP3R complex, was also investigated.

## 2 Results

### 2.1 CPZ Derivatives JX57 and JX66 Exhibit Anticancer Properties in Endometrial Cancer Organoid

An endometrial cancer organoid (ECO) model was created to verify the efficacy of JX57, JX66, and CPZ (Figure 1B). We found that JX57 (IC_50_ = 1.23 ± 0.34 µm) and JX66 (IC_50_ = 6.390 ± 0.87 µm) were more potent than CPZ (IC_50_ = 58.18 ± 7.62 µM) (Figure 1C).

### 2.2 Pharmacokinetic Evaluation of JX57 and JX66

From the perspective of new drug development, good candidates should have excellent pharmacokinetic properties. The plasma protein binding rate of JX57 was 99.16%, while that of JX66 was not available because no peaks were detected in free samples. The compound JX66 may be highly bound or, in some cases, exhibit high nonspecific binding (Table S1, Supporting Information). Comprehensive comparisons of pharmacokinetic parameters showed that JX57 had good oral bioavailability with a value of 94.3%, whereas JX66 only had an oral bioavailability of 2.23% (**Table** **1**). In addition, JX66 did not easily form salts with counterions such as hydrochloric acid, sulfuric acid, phosphoric acid, and citric acid, and its water solubility was poor. The results indicate that JX57 possesses superior pharmacokinetic properties compared with those of JX66.

**Table 1:** Pharmacokinetics (PK) parameters of JX57 and JX66.

PK parameters		JX57	JX66
*T*_max_(h)	PO(10 mg k^−1^g)	0.41 ± 0.14	5.50 ± 4.33
	IP(5 mg k^−1^g)	0.33 ± 0.14	1.67 ± 0.57
*C*_max_(ng/mL)	PO(10 mg k^−1^g)	11.30 ± 4.57	3.80 ± 1.29
	IP(5 mg k^−1^g)	3.04 ± 1.12	10.00 ± 1.49
AUC 0–24 h (h*ng/mL)	PO(10 mg k^−1^g)	41.50 ± 15.70	16.00 ± 2.65
	IP(5 mg k^−1^g)	9.01 ± 7.03	140.00 ± 35.30
	IV(2 mg k^−1^g)	8.12 ± 4.13	144.00 ± 75.90
AUC 24 h^−∞^ (h*ng/mL)	PO(10 mg k^−1^g)	55.7 ± 24.30	NA^a)^
	IP(5 mg k^−1^g)	14.20 ± 8.39	170.00 ± 37.40
	IV(2 mg k^−1^g)	13.90 ± 5.43	153.00 ± 75.90
*T*1/2(h)	PO(10 mg k^−1^g)	4.03 ± 0.88	NA^a)^
	IP(5 mg k^−1^g)	3.06 ± 1.03	10.10 ± 1.38
	IV(2 mg k^−1^g)	2.72 ± 0.90	5.10 ± 1.69
*F*(%)	PO(10 mg k^−1^g)	94.30 ± 46.90	2.23±0.37
	IP(5 mg k^−1^g)	44.40 ± 34.60	42.50 ± 13.00
*CL*(L/h/kg)	IV(2 mg k^−1^g)	159.00 ± 58.7	15.90 ± 8.68

^a)^ There was no specific fitting value, as the peak time for JX66 was 5.5 h, and there were only two data detection points, so the fitting or calculation was not possible.

### 2.3 GRP75 is a Direct Target of JX57 in EC

**Figure 2:**
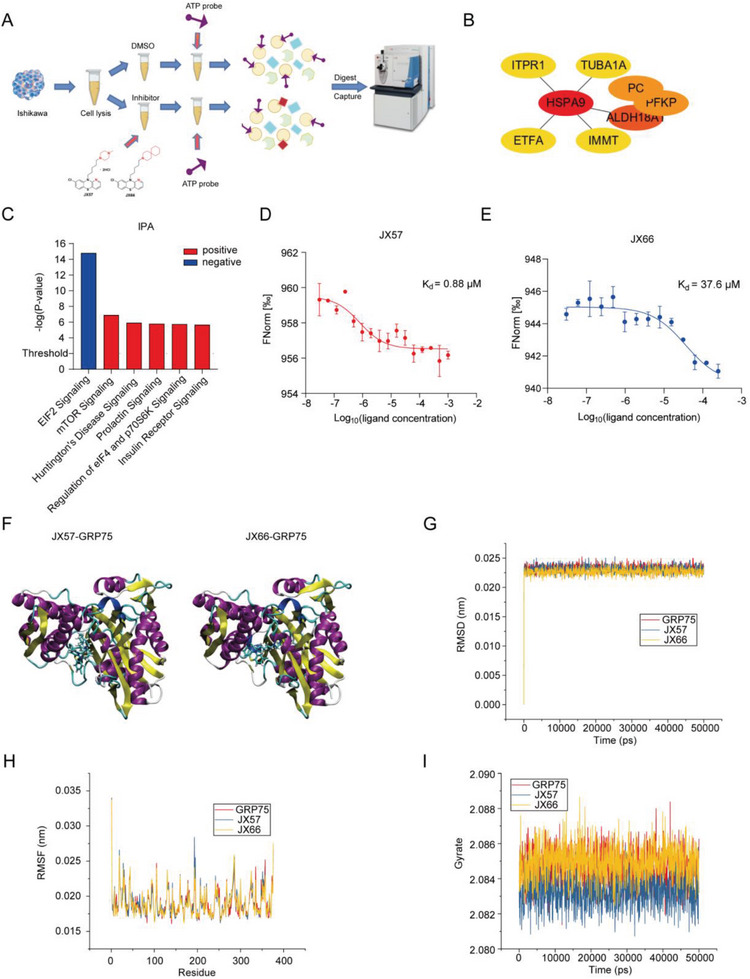
Combining activity‐based protein profiling (ABPP) and transcriptome results identified GRP75 as the direct target of JX57. A) Schematic diagram of the ABPP technology used for the identification of JX57 and JX66 target proteins. B) Protein—protein interaction analysis of candidate targets. Redder colors indicate more central, yellower colors indicate exterior. C) Ingenuity pathway analysis suggests the enrichment pathways that exhibited the most substantial up (positive)‐ and down (negative)‐regulation in EC cells treated with JX57. D,E) Microscale thermophoresis results showing the binding of the GRP75 protein to JX57 and JX66 (concentration units: mol/L); *n* = 3. F) Molecular docking patterns of JX57 and JX66 with GRP75. G) Root mean square deviation of the protein backbone in complex with JX57 and JX66. H) Root mean square fluctuation (RMSF) values of the protein backbone throughout the simulations, where the ordinate is the root mean square fluctuation RMSF (nm) and the abscissa is the residue number. I) Radius of the protein complex gyration profile in the presence of the selected ligands after 50 000 ps molecular dynamics simulations. Data show the mean ± SD.

In conjunction with findings of the preceding investigation,^[^ ^8^ ^]^ JX57 and JX66 exhibited excellent anti‐EC effects in EC cells, ECOs, and mouse xenograft tumors with few extrapyramidal side effects, but their molecular targets were unclear. To investigate this, activity‐based protein profiling determinations were made using a kinase enrichment procedure in the context of EC cell lysate using an adenosine triphosphate (ATP) probe. Then, we isolated and identified some protein factors whose ability to bind ATP appeared modified by the presence of JX57 and JX66 (**Figure** **2**A). These proteins were pulled down by 10% SDS‐PAGE (Figure S1, Supporting Information), and a list of identified proteins is presented in Table S2 (Supporting Information). Interestingly, electron transfer flavoprotein subunit alpha, GRP75, phosphofructokinase, pyruvate carboxylase, and pyrroline‐5‐carboxylate synthase were identified as possible targets of JX57 and JX66. HSPA9 (GRP75) was also identified as the central protein in the protein–protein interaction networks created using the String database and Cytoscape (Figure 2B). The eukaryotic initiation factor 2 (eIF2) pathway was significantly altered according to the results of Ingenuity pathway analysis using sequencing data (Figure 2C; Table S3, Supporting Information). The current study suggests that the eIF2 pathway is closely related to the integrated stress response (ISR), a central regulatory network of signals induced by dysregulation of protein homeostasis,^[^ ^26^ ^]^ which can respond to the heat shock response. These findings suggested that GRP75 is a potential target of JX57 and JX66. To investigate the affinity of JX57 and JX66 for GRP75, the purified GRP75 protein was studied using an *E. coli* expression system (Figure S2, Supporting Information) for a microscale thermophoresis (MST) assay. The results showed that GRP75 could bind to JX57 and JX66 with *K*_d_ values of 0.88 and 37.6 µm, respectively; the binding ability of JX57 was significantly better than that of JX66 (Figure 2D,E). To further investigate the binding pattern between JX57/JX66 and GRP75 (HSPA9), molecular dynamics (MD) simulations were performed for several docked small molecule–protein complexes (Figure 2F; Figure S3, Supporting Information). Root mean square deviation calculation showed that the complexes rapidly reached stable values after the simulation was initialized and then remained stable until the end of the simulation, which indicates that the proteins were ultimately stable in all three systems (Figure 2G). The root mean square fluctuation values of the complexes formed by JX57 or JX66 with GRP75 were essentially identical to those of the original protein residues, indicating that the protein structures remained stable after binding (Figure 2H). Next, we assessed the conformation of GRP75 when complexed with JX57 and JX66. The mean square radius of the gyration characterizes the compactness of the protein, and the smaller the value of this parameter, the more compact the protein structure. Upon the binding of the protein to the JX57 molecule, there is a discernible reduction in the mean‐square radius of the gyration of the resulting complex. This observation implies a modification in the conformation of the protein, leading to a more condensed structure. (Figure 2I). The binding free energy analysis including van der Waals forces, electrostatic forces, polar solvation energy, and solvent accessible surface area (SASA) for JX57 and JX66 (**Table** **2**) showed that JX57 had a better binding ability than JX66 after the MD simulation, likely due to its stronger van der Waals and electrostatic forces.

**Table 2:** van der Waals, electrostatic, polar solvation, SASA, and binding energies of JX57 and JX66 in the binding pocket of GRP75 (Protein Data Bank ID: 4KBO).

System	Energy [kJ mol^−1^]				
	VdW	Elec	Polar solv	SASA	Binding
JX57	−226.603	−62.702	260.674	−20.821	−67.570
JX66	−196.739	−3.073	151.940	−19.698	−49.480

Based on the collected data for the binding of JX57 and JX66 with the potential target GRP75 and the efficacy of both in the ECO model, JX57 was determined more valuable as a new drug candidate and selected for further investigation.

### 2.4 High GRP75 Expression Contributes to Tumorigenesis and Can Predict a Poor EC Prognosis

**Figure 3:**
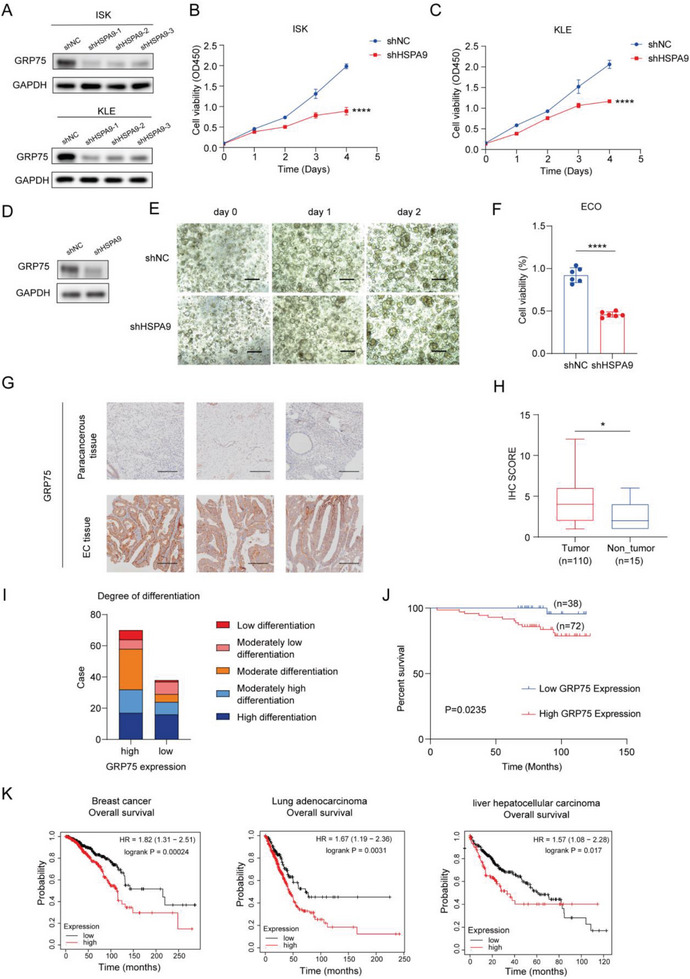
High GRP75 expression contributes to tumorigenesis and correlates with a poor EC prognosis. A) Western blot analysis showing successful knockdown of GRP75 in ISK and KLE cells. B,C) CCK‐8 assays showing the effects of the GRP75 knockdown on EC cell proliferation; *n* = 6. D) Knockdown of GRP75 in ECO cells. E) Representative images of ECO cells; scale bar = 100 µm. F) CCK‐8 assays showing the effects of the GRP75 knockdown on ECO cell proliferation; *n* = 6. G) Representative images of GRP75 staining in the EC tumor and paracancerous tissues; scale bar = 50 µm. H) GRP75 expression in 110 tumor tissues compared with levels in 15 adjacent non‐cancerous tissues. I) High GRP75 expression is correlated with poorer EC differentiation. J) Kaplan–Meier survival analysis of 110 patients with EC based on tumor GRP75 expression. K) GRP75 expression was analyzed with Kaplan–Meier Plotter in patients with breast cancer, lung adenocarcinoma, and liver hepatocellular carcinoma from TCGA database. Data show the mean ± SD. B,C) *p* values were calculated using one‐way ANOVA; F,H) *p* values were calculated using two‐tailed unpaired *t*‐tests; ^*^ *p* < 0.05, ^**^ *p* < 0.01, ^***^ *p* < 0.001, and ^****^ *p* < 0.0001.

The effects of GRP75 knockdown on EC cell proliferation were investigated. GRP75‐deficient stable cell lines were first established (**Figure** **3**A), and the abilities of the GRP75‐knockdown EC cells to proliferate were found to be markedly inhibited (Figure 3B,C). Moreover, the knockdown model was established in the ECOs (Figure 3D), and a decrease in GRP75 expression significantly reduced ECO cell viability (Figure 3E,F).

In the Cancer Genome Atlas database, GRP75 was found to be highly expressed in EC and significantly correlated with stage and survival (Figure S4, Supporting Information). To further study the clinical relevance of GRP75, its expression was evaluated using a tissue microarray containing EC tumor tissues from 110 patients and 15 paracancerous tissue samples using immunohistochemistry analysis. The results showed that the expression of GRP75 in the tumor tissues was higher (4.65) than that in paracancerous tissues (2.80). Moreover, GRP75 expression was significantly associated with tumor differentiation (Figure 3I). Kaplan–Meier survival analysis revealed that the patients with high tumor GRP75 expression exhibited significantly shorter survival than those with lower tumor GRP75 expression (Figure 3J). Analysis of the data from the Kaplan–Meier Plotter confirmed that tumor GRP75 expression could predict the survival of patients with breast cancer, lung adenocarcinoma, and liver hepatocellular carcinoma (Figure 3K).^[^ ^27^, ^28^ ^]^ A correlation analysis showed that GRP75 expression was significantly associated with patient age, differentiation, and survival (Table S4, Supporting Information). Further univariate/multivariate analyses suggested that GRP75 expression may be an independent prognostic factor in EC (Table S5, Supporting Information). A nomogram survival prediction model was established using a Cox regression model analysis to predict the overall survival of patients with EC (Figure S5, Supporting Information). Internal validation of the nomogram model showed a C‐index of 0.792 (SD = 0.126), indicating a good level of consistency between the nomogram‐predicted survival and actual survival. Collectively, these data illustrate that GRP75 promotes tumorigenesis and is a potential prognostic biomarker for EC.

### 2.5 GRP75 is Required for JX57 Bioactivity in Tumorigenesis

**Figure 4:**
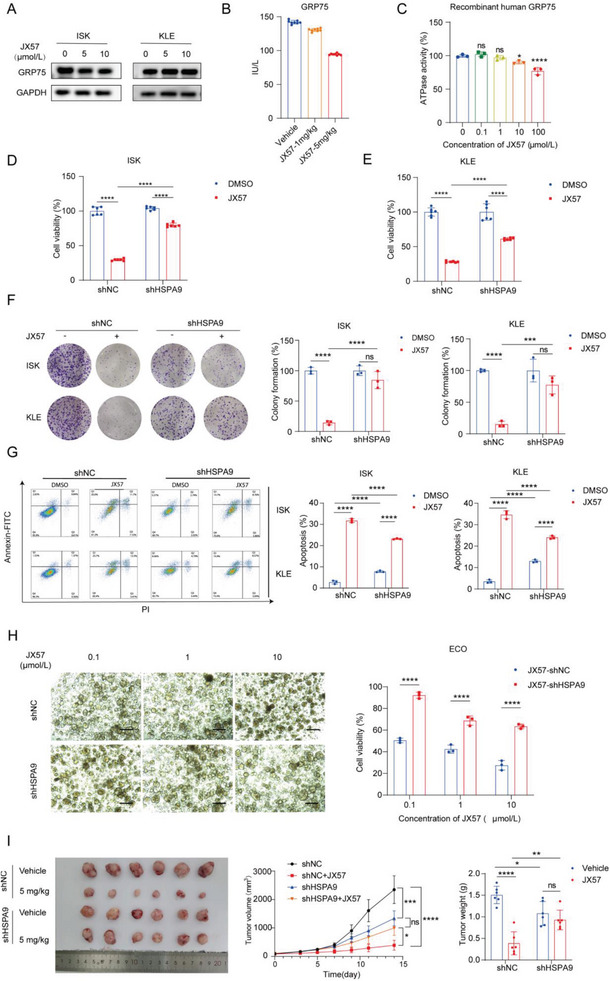
GRP75 is required for the anti‐EC activity of JX57. A) Western blotting analysis of the GRP75 expression in ISK and KLE cells treated with JX57. B) Quantification of GRP75 in JX57‐treated ISK tumor‐bearing mice determined using ELISA; *n* = 8. C) ATPase activity of the recombinant GRP75 treated with gradient concentrations of JX57; *n* = 3. D–G) Cell viability assay; *n* = 6 (D, E), colony formation assay; *n* = 3 (F), and flow cytometry analysis; *n* = 3 (G) to assess the effects of JX57 (10 µmol L^−1^) and DMSO on proliferation and apoptosis in GRP75‐deficient ISK and KLE cells. H) Cell viability assays showing the effects of JX57 on proliferation in GRP75‐deficient ECO cells; scale bar = 100 µm; *n* = 3. I) Harvested tumors at the growth assay endpoint show HSPA9 (GRP75) knockdown inhibits the antitumor effects of JX57; *n* = 6. Data show the mean ± SD. C) *P* values were calculated using one‐way ANOVA; D–I) *p*values were calculated using two‐way ANOVA; ns, not significant; ^*^ *p* < 0.05, ^**^ *p* < 0.01, ^***^ *p* < 0.001, and ^****^ *p* < 0.0001.

To investigate the effect of JX57 on GRP75 expression, the levels of GRP75 protein in cells and tissues were assessed. The results showed that the expression of GRP75 in xenograft tumors with ISK cells was reduced at high doses of JX57 (5 mg kg^−1^), whereas at the cellular level, a slight reduction was observed in ISK cells, but the changes in KLE cells were not significant (**Figure** **4**A,B). Considering GRP75 requires ATP binding and hydrolysis to perform its chaperone function,^[^ ^29^ ^]^ the ATPase enzymatic activity was evaluated using the recombinant human GRP75 protein. JX57 was found to inhibit the ATPase activity of recombinant human GRP75 (Figure 4C).

Cell viability and colony formation analyses showed that the GRP75 knockdown significantly impaired the inhibitory effects of JX57 on EC cell proliferation (Figure 4D–F). Membrane‐linked protein V‐FITC/PI staining showed that the silencing of GRP75 significantly attenuated the effects of JX57 on EC cell apoptosis (Figure 4G). Furthermore, the efficacy of JX57 was diminished in organoids with GRP75 knockdown, thereby providing further evidence that GRP75 serves as a target for JX57 to elicit anticancer properties. (Figure 4H). To examine the role of GRP75 in EC tumorigenesis and the antitumor activity of JX57 in animals, GRP75‐deficient ISK cells were subcutaneously injected into the armpits of nude mice. The GRP75‐deficient mice showed lower tumor volume and tumor weight (42.9% and 28.4% lower, respectively) than the controls, and the effects of JX57 on the knockdown tumor were not significantly different from those in the vehicle group (Figure 4I).

### 2.6 JX57 Disrupts MAM Integrity by Inhibiting the GRP75–IP3R Complex

**Figure 5:**
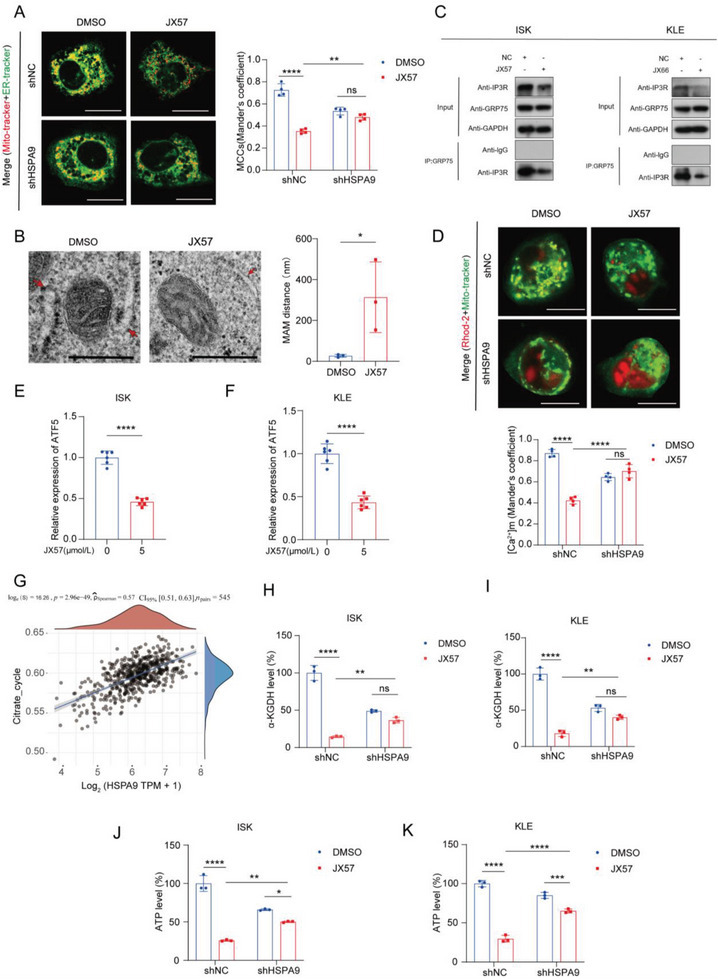
JX57 destabilizes the MAM structure via GRP75. A) Representative confocal images of ISK cells and HSPA9 (GPR75) knockdown cells treated with JX57 (10 µmol L^−1^) and stained with Mito‐Tracker (red) and ER‐Tracker (green); scale bar = 20 µm. Manders’ co‐localization coefficient (MCC) quantification of the overlap between Mito‐Tracker and ER‐Tracker; *n* = 4. B) Representative TEM images of the MAM in ISK cells that were treated with DMSO and JX57 (10 µmol L^−1^). Red lines with arrows indicate the contact distance for the Mito–ER associations; scale bar = 500 nm. Quantification of the mean distance between the Mito and ER associations; *n* = 3. C) Co‐immunoprecipitation analysis of GRP75 and IP3R in the ISK cells and KLE cells treated with JX57 (10 µmol L^−1^). D) Representative confocal images of Rhod‐2 (red)‐ and Mito‐Tracker (green)‐loaded ISK cells and HSPA9 knockdown cells treated with JX57 (10 µmol L^−1^); scale bar = 20 µm. Quantification of the [Ca^2+^]_m_ levels (MCC of Rhod‐2 signal overlapping with Mito‐Tracker signal); *n* = 4. E,F) mRNA expression of ATF5, detected by quantitative real‐time polymerase chain reaction; *n* = 6. G) Correlations between HSPA9 and TCA were cycle scores analyzed using the Spearman correlation. The abscissa represents the distribution of gene expression, and the ordinate represents the distribution of the pathway score. H,I) JX57 (10 µmol L^−1^) reduces α‐KGDH levels as mediated by GRP75 in the ISK and KLE cells; *n* = 3. The α‐KGDH level in the shNC group was set at 100%. J,K) JX57 (10 µmol L^−1^) reduces ATP levels as mediated by GRP75 in ISK and KLE cells; *n* = 3. The ATP levels in the shNC were set at 100%. Data show the mean ± SD. B,E,F) *p* values were calculated using two‐tailed unpaired *t*‐tests; A,D,H–K) *p* values were calculated using two‐way ANOVA; ns, not significant; ^*^ *p* < 0.05, ^**^ *p* < 0.01, ^***^ *p* < 0.001, and ^****^ *p* < 0.0001.

We hypothesized that the bridging role played by GRP75 in MAM structure is key to JX57 targeting and anticancer effects. In order to examine the validity of this hypothesis, we conducted a sequencing analysis on ISK cells with GRP75 knockdown and integrated the resulting data with previously obtained transcriptome data from JX57‐treated ISK cells. Our analysis revealed a significant enrichment of pathways related to Ca ion transport and metabolism, aligning well with the established role of GRP75 in MAM structures (Figure S6, Supporting information). The ER–mitochondrial association was found to be reduced in EC cells after JX57 treatment, and this reduction was attenuated by the knockdown of GRP75 (**Figure** **5**A). Additionally, transmission electron microscopy (TEM) revealed that JX57 significantly expanded the ER–mitochondrial distance in EC cells, indicating that JX57 may compromise the structural integrity of the MAM in EC cells (Figure 5B). These results suggest that JX57 impairs MAM integrity in EC cells by targeting GRP75.

Given that the IP3R–GRP75 complex plays an important role in the calcium input between the ER and mitochondria and the structural formation of the MAM, the GRP75–IP3R interaction in the MAM was evaluated. Co‐immunoprecipitation showed that JX57 attenuated the interactions between the two components (Figure 5C). Confocal imaging was used to further examine the effects of JX57 on the ER in relation to mitochondrial Ca^2+^ transfer. The results showed that JX57 decreased the [Ca^2+^]_m_ levels in EC cells, and this effect was attenuated with the knockdown of GRP75 (Figure 5D).

Agonist‐induced IP3R Ca^2+^ signaling promotes OXPHOS and ATP production mainly through the stimulation of tricarboxylic acid (TCA) cycle dehydrogenases (PDH, α‐KGDH) and respiratory chain components.^[^ ^30^, ^31^ ^]^ Considering that JX57 triggered IP3R degradation, alterations in the processes related to mitochondrial bioenergetics were evaluated. Reverse transcription quantitative PCR (RT‐qPCR) showed that JX57 decreased the transcript‐level expression of activating transcription factor 5 (ATF5), which is known to play an important role in mitochondrial biogenesis (Figure 5E,F).^[^ ^32^ ^]^ A set of genes in the TCA cycle pathway was identified, and according to the ssGSEA algorithm, the enrichment scores showed a significant positive correlation between HSPA9 (GRP75) and the TCA cycle (Figure 5G). Furthermore, analysis of the intracellular ATP levels and the enzymatic activity of key TCA cycle enzymes revealed that JX57 reduced the cellular ATP levels and α‐ketoglutarate activity, and this was attenuated in the GRP75 knockdown cell lines (Figure 5H–K). These results suggest that JX57 dysregulates mitochondrial calcium homeostasis by inhibiting GRP75, which leads to mitochondrial dysfunction in EC cells.

### 2.7 JX57 Triggers a Bioenergetic Crisis to Exert Anticancer Activity in EC Cells

**Figure 6:**
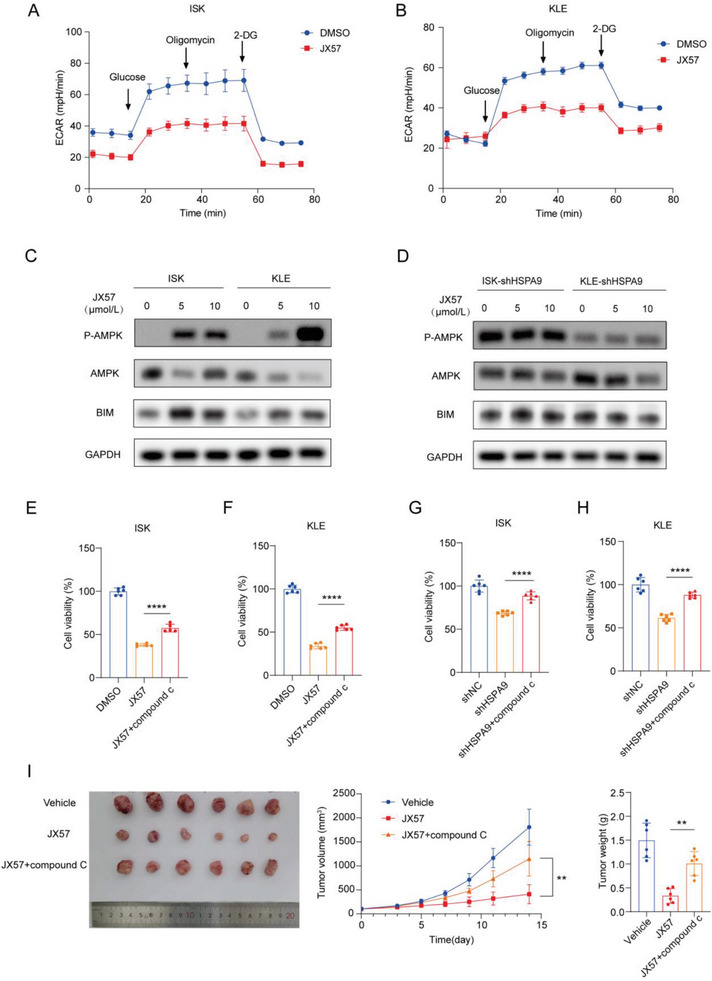
JX57 acts on GRP75 to activate AMP‐activated protein kinase (AMPK) signaling in EC cells to exert anticancer effects. A,B) Extracellular acidification rate (ECAR) was measured using the Seahorse XFe96 analyzer in ISK and KLE cells; *n* = 6. C) Expression of p‐AMPK, AMPK, and BIM determined by Western blotting in ISK and KLE cells treated with JX57. D) Western blotting of HSPA9‐deficient ISK and KLE cells treated with JX57. E) ISK and F) KLE cells were treated with JX57 (10 µmol L^−1^) for 48 h in the presence or absence of compound C (0.5 µmol L^−1^), and a CCK‐8 assay was used to measure cell viability; *n* = 6. G) ISK and H) KLE cells were treated with compound C for 48 h (0.5 µmol L^−1^), and a CCK‐8 assay was used to measure cell viability; *n* = 6. I) Harvested tumors at the growth assay endpoint show the antitumor effects of JX57 (5 mg kg^−1^) when compound C (0.2 mg kg^−1^) was added; *n* = 6. Data for the glycolysis experiment are shown as mean ± SEM and other data are shown as mean ± SD. E–I) *p* values were calculated using one‐way ANOVA;^*^ *p* < 0.05, ^**^ *p* < 0.01, ^***^ *p* < 0.001, and ^****^ *p* < 0.0001.

To further verify the effects of JX57 on bioenergy and mitochondrial function, we determined the effects of JX57 on glycolysis. A Seahorse glycolytic rate assay was performed using ISK and KLE cells treated with JX57 (**Figure** **6**A,B). JX57 markedly reduced the glycolysis rate and glycolytic capacity of the ISK and KLE cells, as reflected by the extracellular acidification rate (ECAR), and exerted an effect on oxidative phosphorylation, as indicated by the oxygen consumption rate (OCR) (Figure S7, Supporting Information). Considering the impact of the JX57 on energy homeostasis and the enrichment of the AMPK pathway in the GRP75‐knockdown cells (Figure S7, Supporting Information), AMPK signaling, a well‐recognized cellular energy sensor that can be activated by energy stress, may play an important role in the anticancer bioactivity of JX57. Given that BIM may be a downstream apoptotic molecule for AMPK to exert anticancer effects,^[^ ^33^, ^34^ ^]^ we postulated that GRP75/AMPK/BIM is necessary for JX57 to exert its anticancer activity. Western blotting confirmed that JX57 increased p‐AMPK expression and BIM expression in ISK and KLE cells, and this trend was weakened in GRP75‐deficient cells (Figure 6C,D). The caspase activity test also confirmed the effects of the GRP75 on apoptosis (Figure S8, Supporting Information). To determine whether the AMPK pathway is responsible for the anticancer activity of JX57 via GRP75 in EC, the effect of compound C (AMPK inhibitor) on the anticancer effect triggered by JX57 and GRP75 knockdown was examined. Data from the cell counting kit 8 (CCK‐8) assay showed that AMPK inhibition significantly decreased the inhibition of proliferation in the JX57‐treated EC cells and GRP75‐deficient EC cells (Figure 6E–H). More importantly, we found that the addition of the AMPK inhibitor partially abolished the anticancer effect of JX57 in vivo (Figure 6I).

## 3 Discussion

Emerging evidence suggests that drug repositioning is a fast and effective strategy to identify new purposes and functions for existing drugs. Recent studies have indicated that FDA‐approved drugs, including antipsychotics, may be promising candidates for drug repurposing.^[^ ^35^ ^]^ Epidemiological studies conducted on a large cohort of patients with schizophrenia showed a reduced incidence of cancer in those patients undergoing psychosis treatment.^[^ ^36^, ^37^ ^]^ Various dopamine receptor D2 antagonists have been tested in cancer cells and found to target tumor immunity, cell invasion, and migration in cancer stem‐like cells.^[^ ^38^, ^39^ ^]^ Considering the possible benefits of antipsychotics in cancer treatments, the repurposing of these medications to treat one or more specific cancer (sub)types could be a rapid pathway to clinical translation.^[^ ^40^ ^]^ CPZ, which belongs to the phenothiazine class of antipsychotic drugs, was identified as a potential candidate for drug repurposing in cancer treatment, specifically for EC.^[^ ^7^, ^36^ ^]^ It has been reported to have anti‐proliferative effects, as it alters the expression of proteins that regulate the cell cycle and intracellular mitochondrial functions.^[^ ^41^, ^42^, ^43^, ^44^ ^]^ However, the repurposing of CPZ for cancer therapy is challenging, as CPZ is a first‐generation antipsychotic that also has some serious limitations, such as extrapyramidal symptoms.^[^ ^45^ ^]^ To address this issue, JX57 was developed by structural optimization and found to produce fewer side effects than CPZ.^[^ ^8^ ^]^ In this study, the direct target and mechanisms of JX57 were explored to provide new insights and offer guidance for future research into the mechanisms of antipsychotics and their anticancer effects.

GRP75 was identified as a direct target that mediates the anticancer effects of JX57. As a member of the Hsp70 family, GRP75 plays an important role in cancer progression,^[^ ^46^, ^47^ ^]^ but its biological functions and clinical significance are currently unknown. Here, we report for the first time that GRP75 is frequently upregulated in EC tissues, and could thus be a predictor of poor prognosis for EC patients (Figure 3). In vitro and in vivo functional assays supported the idea that GRP75 contributes to tumorigenesis in EC. JX57 was also confirmed to directly bind to GRP75 (Figure 2) through activity‐based protein profiling and MST assays. More importantly, a series of functional assays, both in vitro and in vivo, showed that the knockdown of GRP75 could reduce the anticancer biological activity of JX57 (Figure 4). These results provide the first evidence that GRP75 may be a potential therapeutic target in EC. Future studies targeting the clinical significance of GRP75 will be of great value, and GRP75 protein expression could be a useful biomarker by which to stratify patients with EC for individualized therapies.

Heat shock proteins (HSPs) are rational targets used for the development of new cancer therapeutics, as cancer cells depend on the increased levels of HSPs to maintain their unstably elevated proliferative and metabolic states.^[^ ^48^ ^]^ The transport of Ca^2+^ from the ER to the mitochondria is mediated by the IP3R‐GRP75 complex in the MAM structure, which is critical for maintaining cellular bioenergy. IP3R induces bioenergetic stress leading to reduced oxidative respiration, which inhibits the TCA cycle process.^[^ ^49^ ^]^ In this study, an anticancer mechanism for disrupting the IP3R–GRP75 complex was found to facilitate MAM integrity and thus reduce the calcium inputs from the ER to the mitochondria, thereby dysregulating mitochondrial energy homeostasis, which could indicate a new anticancer mechanism involving GPR75 inhibition. Our results help to elucidate the mechanism of the anticancer effects of JX57 targeting GRP75 and establish the importance of the dysregulation of mitochondrial calcium homeostasis for cancer cell survival.

The nervous system and cancer interact in a variety of ways. Drugs for the central nervous system may also affect tumors by interacting with neurotransmitters. The anticancer activity of JX57, a derivative of the dopamine receptor D2 antagonist CPZ, could result from its effects on the nervous system and tumor associations; however, in this study, only the direct target of action on tumor cells was assessed, representing a limitation of the study. In clinical practice, this direct effect could be achieved through local injection of the JX57 by uterine artery intervention. However, whether JX57 can indirectly act on the EC through the nervous system at the same time should be further investigated to overcome this limitation.

## 4 Conclusion

In summary, we have reported for the first time that GRP75 contributes to EC progression and is required for the anticancer bioactivity of the CPZ derivative JX57, both in vitro and in vivo. The CPZ derivative JX57 was found to act directly on EC via GRP75 without relying on the indirect effects of the nervous system. JX57 suppresses EC progression by inhibiting the IP3R–GRP75 complex, which disrupts the structural integrity of the MAM and triggers a mitochondrial energy crisis. Taken together, our findings suggest that GRP75 may be a promising therapeutic target and prognostic biomarker for EC.

## 5 Experimental Section

### Cell Culture

Human endometrial carcinoma cell lines (ISK and KLE) were obtained from the American Type Culture Collection (Manassas, VA, USA) and routinely maintained in complete medium (Dulbecco's modified Eagle's medium [DMEM]/F12 [HyClone, SH30271.01; Cytiva Marlborough, MA, USA] supplemented with 10% fetal bovine serum [Gibco, 16000–044; Thermo Fisher Scientific, Waltham, MA, USA], and 1% penicillin–streptomycin [HyClone, SV30010]) in a humidified incubator at 37 °C with 5% CO_2_.

### ECO Culture

The study was approved by the International Peace Maternity and Child Health Hospital Ethics Committee, School of Medicine, Shanghai Jiaotong University, Shanghai, China ([GKLW] 2017–125). Research participants, all of whom provided written consent, were recruited from the International Peace Maternity and Child Health Hospital. Only leftover tissues from the hysterectomy specimens obtained from women with endometrial endometrioid carcinoma were utilized.

ECO cultures were established as described previously.^[^ ^50^ ^]^ Biopsies were cut into small pieces and extensively rinsed in phosphate‐buffered saline (PBS; HyClone, SH30256.01) containing 2% penicillin–streptomycin solution (Thermo Fisher Scientific, 10 010 023). Tissues were incubated in TrypLE (1x; Thermo Fisher Scientific, 12 604 013) supplemented with rock inhibitor for 20 min and then mechanically sheared to obtain a cell suspension. Digestion was stopped by dilution with equal amounts of DMEM/F12 and centrifuged at 1300 rpm for 5 min at 4 °C. The pellet was resuspended in Matrigel (Corning, 356 231, Corning, NY, USA), and 30 µL droplets were deposited in pre‐warmed 24‐well plates. ECOs were cultured as described previously.^[^ ^51^ ^]^ For passages (performed every 10–20 d), organoids were recovered via liquefaction of the Matrigel drops with ice‐cold DMEM/F12 to ensure maximum organoid collection. Subsequently, ECOs were dissociated by repeated pipetting using TrypLE (in DMEM/F12 containing rock inhibitor), and the mixture was centrifuged at 1300 rpm. The resulting cell suspension was digested and pelleted, and organoids were recovered, as described above. The established organoids were expanded and cryopreserved for biobanking.

### Tumor Xenograft Experiments

All animal experiments were approved by the Committee on the Ethics of Animal Experiments of Shanghai Jiao Tong University, China (GKLW 2018–40). Female BALB/c nude mice (at least 5 weeks old) were procured from a commercial supplier (JieSiJie, Shanghai, China) through the Department of Laboratory Animal Science at Shanghai Jiaotong University School of Medicine. Mice were maintained under standard conditions and cared for according to the institutional guidelines for animal care. EC cells resuspended in PBS were mixed with Matrigel (BD Biosciences) in equal volume and then subcutaneously injected into the armpits of the nude mice. When the tumor diameter reached ≈6 mm, the mice were randomly grouped (at least six mice per group) and received a daily intraperitoneal (i.p.) injection of the vehicle (dimethylsulfoxide [DMSO], 200 µL 20 g^−1^), JX57 (5 mg kg^−1^), or compound C (0.2 mg kg^−1^). The dose of the compound injected into the mice was slightly adjusted following the methodology described in the previous study.^[^ ^7^ ^]^ Every 2 d, the length (*L*) and width (*W*) of each tumor were measured twice using an external Vernier caliper for automatic reading, where the larger of the two measurements was defined as *L* and the smaller as *W*. The tumor volume was calculated using Equation 1, as follows:

(1) $$V=0.52\times \left(L\times W^{2}\right)$$

### Clinical Sample Collection

EC and paracancerous tissue microarrays were purchased from Superbiotek (EMC1351). A total of 125 tissue samples were obtained, including 110 EC cancer tissues and 15 adjacent tissues.

### Pharmacokinetic Assay

Three male SD rats (208–259 g, 6–8 weeks) per group were purchased from JH Laboratory Animal Co. LTD. All rats were raised under controlled temperature and humidity conditions and had free access to food and water. Compounds (JX57, JX66, and CPZ) were dissolved in 5% DMSO and diluted with 8% castor oil and 87% PBS solution to a stock concentration of 20 mg mL^−1^. Each rat was injected with a combination solution at doses of 2, 10, and 5 mg kg^−1^ for IV, PO, and IP, respectively. Blood samples collected in anticoagulant (K2EDTA)‐containing tubes at 0.25, 0.5, 1, 2, 4, 8, and 24 h after injection were centrifuged at 2000 × *g* at 4 °C for 5 min to obtain plasma. LC‐MS/MS analysis of samples was carried out using an ACQUITY UPLC HSS T3 1.8 µm column. The mobile phase was a mixture of phase A (0.1% formic acid in water) and phase B (0.1% formic acid in acetonitrile), which ran in gradient mode at a flow rate of 0.6 mL min^−1^ at 60 °C. Mass spectra were obtained on an API6500 triple quadrupole equipped with an ESI source. Glipizide was used as an internal standard. The plasma concentrations of the compounds were analyzed, and the pharmacokinetic parameters were calculated via WinNonlin.

### Tissue Microarray and Immunohistochemistry

Immunohistochemistry was performed as described previously.^[^ ^52^ ^]^ The expression of GRP75 was analyzed in the tissue microarray samples. In brief, the tissue microarray was incubated with GRP75 antibody (1:100 dilution, Santa Cruz Biotechnology, Dallas, TX, USA) overnight at 4 °C followed by a corresponding biotinylated secondary antibody. For scoring, each microarray core was categorized into two groups: score < 4 (low), and score ≥ 4 (high).

### Enzyme‐Linked Immunosorbent Assay (ELISA)

HSP‐70 ELISA kits were purchased from YuBo Biology. The samples were obtained from the mouse tumor tissues treated with JX57 in previously reported efficacy experiments.^[^ ^8^ ^]^ Equal amounts of diluted protein supernatant were used, and the assay was conducted according to the manufacturer's instructions. The obtained absorbance values for protein levels were then transferred to in‐house data analysis software developed using the Windows Microsoft Excel platform to calculate the standard deviations for each group, and the average absorbance for each group was compared to the standardized controls. Each test group for each of the proteins of interest consisted of eight replicates. Assay‐specific controls were run with each plate to ensure optimal accuracy of the results.

### CCK‐8 Assay

EC cells were seeded into 96‐well plates at a concentration of 8 × 10^3^ cells well^−1^ in a 100 µL volume and cultured overnight. Cells were then treated with various concentrations (0.01, 0.1, 1, 10, 100 µm) or fixed concentrations (10 µm) of compounds to test their viability. Following treatment for 48 h, the supernatant was removed, and 100 µL of CCK‐8 (Yeasen, Shanghai, China) solution (1:10 dilution in complete medium) was added to each well and incubated in the dark for 1 h at 37 °C. Absorbance at 450 nm was measured using a microplate reader (BioTek Synergy H1, Agilent Technologies, Santa Clara, CA, USA).

### Colony Formation Assay

EC cells (500 per well) were seeded into six‐well plates and cultured overnight (37 °C, 5% CO_2_). The following day, JX57 (10 µm) was diluted with complete medium and added to each well. The cells were then incubated with the designated drugs, and the medium was renewed every 3 d until cell colonies were visible to the naked eye for 10 d. At the end point, cell colonies were rinsed twice with cold PBS, fixed with precooled anhydrous methanol (Sigma–Aldrich, St. Louis, MO, USA), stained with 0.5% crystal violet solution (Yeasen), and finally rinsed gently under tap water. A high‐resolution camera (Leica, Wetzlar, Germany) was used to acquire full‐field images of the 6‐well plates after drying. ImageJ software (developed by the National Institutes of Health, USA) was then used to count the colonies.

### Apoptosis Assay

For apoptosis analysis, EC cells were incubated with 10 µm of JX57 for 48 h. Following the collection of the cell supernatant, cells were trypsinized and centrifuged. Harvested cells were then washed twice with PBS and stained for 15 min with Annexin V‐FITC/PI (Yeasen). The stained cells were evaluated using flow cytometry (BD Bioscience, Franklin Lakes, NJ, USA).

### Gene Pathway Correlation Analysis

RNA sequencing expression (level 3) profiles and corresponding clinical information for EC were downloaded from the TCGA dataset (https://portal.gdc.com). The R software GSVA package with method “ssgsea” was used for the analysis. The correlation between genes and pathway scores was analyzed using the Spearman correlation. The analyses were all performed using R version 4.0.3, and a *p* value <0.05 was considered statistically significant.

### ATPase Activities of GRP75

The ATPase activities of GRP75 were measured following established protocols.^[^ ^53^ ^]^ Briefly, ATPase activity assays were conducted using the recombinant GRP75 protein. JX57 (0.1, 1, 10, 100 µm) was added to the protein solution in a 96‐well plate. After 30 min of incubation at room temperature (25–28 °C), 10 mm ATP was added to each well, and the plates were further incubated for 3 h at 37 °C. An ATPase assay kit (Elabscience, Wuhan, China) was used to determine ATPase activity, according to the manufacturer's protocols.

### Isolation of MAM Fractions from EC Cells

MAM fractions were isolated following established protocols.^[^ ^54^, ^55^ ^]^ Briefly, the nuclei and unbroken cells were pelleted twice by centrifugation at 600 × *g* for 5 min. The supernatant was then collected and centrifuged at 7000 × *g* for 20 min to isolate the crude mitochondria from the microsomal and ER fractions. After two washes, the crude mitochondria fraction was suspended in 2 mL of mitochondrial resuspension buffer (pH 7.4), layered on 30% Percoll medium, and centrifuged at 95 000 × *g* for 30 min.

### ATP Production and α‐KGDH Activity

EC cells were cultured (2 × 10^5^ well^−1^) and treated with or without JX57 (10 µm). The intracellular ATP levels were then checked using an enhanced ATP kit (Beyotime Biotechnology, Jiangsu, China), in accordance with the manufacturer's instructions. The entire cell population, including any floating cells, was collected, lysed, and boiled to release the ATP for the assay, and the results were compared with those from the DMSO group (%).

Intracellular α‐KGDH activity levels were measured using an α‐KGDH activity assay kit (Solarbio, Beijing, China) according to the manufacturer's instructions. Briefly, the entire cell population, including any floating cells, was collected, milled, and the absorbance measured. The α‐KGDH activity was then determined using Equation 2 as follows:

(2) $$\alpha -KGDH=U/10^{4}cell=4.962\times \left(\Delta A_{\mathrm{determination}}-\Delta A_{\mathrm{blank}}\right)$$

The results were compared to those from the DMSO group (%).

### RNA Sequencing and Ingenuity Pathway Analysis

ISK cells were divided into eight Petri dishes and incubated with DMSO (control) or JX57 (10 µmol L^−1^) for 48 h, with four plates per treatment. The cells were then rinsed twice with PBS, and 1 mL TRIzol (Thermo Fisher Scientific) was added to each dish to collect the total RNA. The Illumina TruSeqTM RNA Sample Prep Kit (Illumina, San Diego, CA, USA) was used for library construction, and the Illumina NovaSeq 6000 sequencing platform was used for the sequencing experiment, according to the manufacturer's instructions. Based on the quantitative expression results, DESeq2 was used to obtain differentially expressed genes between the control group and the JX57 group. The screening threshold was |log2FC| ≥ 0.585 and adjusted *p* < 0.05. Compared with the control group, the differentially expressed genes (fold change > 2.0) were subjected to Ingenuity pathway analysis (Ingenuity Systems, Redwood City, CA, USA) for pathway enrichment.

### MST Assay

To evaluate the binding affinity of JX57 and GRP75, an MST assay was conducted using Monolith NT Automated (NanoTemper Technologies, München, Germany). Purified GRP75 protein was obtained by Zoonbio Biotechnology Co., Ltd. (Nanjing, China). Recombinant His‐tagged GRP75 protein was labeled with RED‐tris‐NTA second generation dye solution (MO‐L018, NanoTemper Technologies), according to the manufacturer's instructions. The concentration of the final labeled GRP75 concentration was 50 nmol L^−1^; GRP75 was mixed with different concentrations of JX57 and JX66 by pipetting multiple times. All samples were diluted in 1× PBST and contained the same amount of DMSO. The *K*_d_ was determined in MO. Control using the *K*_d_ fit.

### MD Simulations

MD simulations for several docked complexes were performed separately using the Gromacs 2020.6 software package.^[^ ^56^ ^]^ The Amber99SB‐ILDN force field was chosen for the proteins,^[^ ^57^ ^]^ while the general Amber force field was generated using the acpype tool for all small molecules of the ligands.^[^ ^58^ ^]^ The spc216 water molecule model was used to fill the complex box. The entire simulation process was divided into four steps: first, 5000 steps for steepest descent optimization; followed by 5000 steps for conjugate gradient optimization to obtain the lowest energy structure; followed by 1000 ps of pre‐equilibration at constant 298.15 K in NPT synthesis; and finally, 50 000 ps at a constant pressure of 1 bar and constant temperature of 300 K using NPT synthesis simulations. To calculate the binding energies of the ligand and protein and the energy decomposition, the g_mmpbsa program was used as described previously.^[^ ^59^, ^60^ ^]^ The last 10 ns of the trajectory were used for analysis; 100 snapshots were taken at each 100 ps node, and the binding energies of the protein and ligand were calculated and energy decomposition analysis was conducted using the MM/PBSA method.

### Western Blotting and Co‐Immunoprecipitation

EC cells were lysed in RIPA buffer (Epizyme, PC102, Ipsen Biopharmaceuticals, Cambridge, MA, USA) supplemented with a protease inhibitor cocktail. Samples were then quantified using a BCA kit (Epizyme, ZJ101), resolved by SDS‐PAGE, and blotted with primary antibodies and corresponding horseradish peroxidase‐conjugated secondary antibodies. The primary antibodies used included anti‐AMPK (1:1000 dilution), anti‐p‐AMPK (1:1000 dilution), anti‐BIM (1:1000 dilution), and anti‐GRP75 (1:1000 dilution) from Cell Signaling Technology (Beverly, MA, USA) and anti‐GAPDH (1:1500 dilution) from Yeason.

Co‐immunoprecipitation of the MAM fractions was conducted using Protein A/G PLUS‐Agarose (Santa Cruz Biotechnology, sc‐2003). Isolated MAM pellets were resuspended in 0.1% NP40 lysis buffer. For pre‐clearing, 1 µg of the control IgG was incubated with 20 µL volumes of the beads and incubated with MAM lysate at 4 °C for 30 min. The lysate was centrifuged at 4 °C for 5 min at 1000 × *g*, and the supernatant was incubated with the antibodies at 4 °C overnight. After adding 20 µL of the resuspended beads at 4 °C for 30 min, the collected beads were washed, and the precipitated protein was examined by Western blotting.

### RT‐qPCR

Total RNA was extracted using Trizol (Thermo Fisher Scientific) according to the manufacturer's instructions. Reverse transcription was performed using PrimeScript II First Strand cDNA Synthesis Kit (TaKaRa Bio, Kusatsu, Japan).^[^ ^61^ ^]^ qPCR was performed using SYBR Premix Ex TaqII (TaKaRa Bio) on a MiniOpticon Real‐Time PCR system (Bio‐Rad Laboratories, Hercules, CA, US). The primers used for ATF5 were 5′‐ CCTTCACCCTCCCGACC‐3′ (forward) and 5′‐ CAGAGTCATGTGGAACGGGA‐3′ (reverse).

### ATP Probe‐Based Drug Profiling

EC cell lysates were prepared and labeled according to the manufacturer's recommendations for the Pierce Kinase Enrichment Kit and ActivX Probes (Thermo Fisher Scientific).^[^ ^62^, ^63^ ^]^ Briefly, 1 × 10^7^ cell pellets were resuspended in 600 mL of affinity purification buffer and sonicated. The lysates were cleared by centrifugation at 16 000 × *g* for 10 min at 4 °C and desalted using Zeba Spin Desalting Columns. The protein concentration was measured using a Bradford assay, and a total of 1 mg was used for drug profiling per sample. MnCl_2_ was added to the lysate to a final concentration of 20 mmol L^−1^ 10 min prior to the drug treatment. The final concentration of the drug for drug profiling was determined using a cell viability assay. After 15 min of lysate preincubation with the drug and DMSO as the control, ATP probe competition reactions were performed at a final concentration of 5 mmol L^−1^ of the desthiobiotin‐ATP probe for 15 min. All reactions were performed at room temperature (25–28 °C) in duplicate.

### MS Sample Preparation and LC/MS‐MS Analysis

The volatile dry peptide samples were redissolved in Nano‐HPLC Buffer A. Separation was performed using the Nano‐HPLC Liquid Phase System UltiMate 3000 RSLCnano (Thermo Fisher Scientific). Liquid phase A was a 0.1% formic acid–water solution, and liquid phase B was a 0.1% formic acid–acetonitrile solution. The chromatographic trap column (100 µm × 20 mm; RP‐C18, Agilent Technologies) was equilibrated with 100% A solution at 3 µL min^−1^. Samples were loaded using an autosampler, combined on the trap column, and then separated by an analysis column that was 75 µm × 150 mm (RP‐C18, New Objective, Littleton, MA, USA), at a flow rate of 300 nL min^−1^. Samples were washed once with a 30 min mobile phase gradient, with a blank solvent between samples. The enzymatic products were separated using capillary HPLC and analyzed using mass spectrometry with a Q‐Exactive Plus mass spectrometer (Thermo Fisher Scientific).

### TEM Analysis

EC cells were fixed with 2.5% glutaraldehyde in sodium cacodylate buffer (0.1 mol L^−1^, pH 7.2) at 4 °C overnight and post‐fixed for 1 h in 1% osmium tetroxide. Samples were stained for 1 h with 1% uranyl acetate in water before dehydration and embedding in the TAAB resin. Images were obtained using a transmission electron microscope.

### Co‐Localization Analysis of ER, Mitochondria, and Ca^2+^ Probes

Co‐localization analysis of the ER and mitochondria was conducted as previously described.^[^ ^64^, ^65^ ^]^ EC cells grown on glass slides were loaded with Mito‐Tracker Red (0.3 µmol L^−1^) and ER‐Tracker Green (1 µmol L^−1^) at 37 °C for 30 min. Images were captured using a confocal microscope equipped with a 100× oil immersion objective (excitation at 488 and 594 nm, emission at 505–530 and >560 nm, respectively). Co‐localization of the ER and mitochondria was quantified as Manders’ co‐localization coefficient (MCC) using ImageJ software with a JACoP plugin. MCCs were calculated according to the fraction of mitochondrial pixels in contact with the ER (with a higher value representing more co‐localization). The co‐localization of Rhod‐2 (red fluorescent signal, 4 µmol L^−1^) with Mito‐Tracker Green (0.3 µmol L^−1^) in the live cells was performed as described above.

### Cell Transfection

The construction of recombinant adenovirus vector systems for GRP75 knockdown was performed by OBiO Technology (Shanghai, China). EC cells were seeded into six‐well culture plates with 1 × 10^5^ cells per well in the corresponding culture medium plus 10% fetal bovine serum. After adherence, cells were transfected by adding the adenovirus particles to the culture at a multiplicity of infection of 30 for 24 h. For the Western blot assay, cells were cultured in fresh medium for 24 h. The recombinant adenovirus containing GRP75 short hairpin RNA (shRNA) or negative control shRNA (shNC) was packaged using the pCLenti‐U6‐shRNA‐CMV‐Puro‐WPRE vector. The shRNAs for GRP75 were: shRNA‐1, CCGAGTCAGATTGGAGCATTT; shRNA‐2, GCTGTCACCAACCCAAACAAT; and shRNA‐3, GCACATTGTGAAGGAGTTCAA. The shNC was TTCTCCGAACGTGTCACGT.

### Seahorse Assay

The ECAR and OCR were measured using the Seahorse XF Glycolysis Stress Test Kit or Seahorse XF Cell Mito Stress Test Kit on the Seahorse XF 96 Extracellular Flux Analyzer (Seahorse Bioscience, North Billerica, MA, USA), according to the manufacturer's introductions. In brief, 1 × 10^4^ EC cells were plated on XF96 cell culture microplates and treated with JX57 (10 µM). Glucose, oligomycin, and 2‐deoxy‐glucose were used to determine the ECAR values, whereas oligomycin, carbonyl cyanide‐p‐trifluoromethoxyphenylhydrazone, and rotenone were used to measure the OCR.

### Statistical Analysis

The data in Figure 3F; Figure 4C,D–F,H; Figure 5H–K; and Figure 6C–H were preprocessed and normalized to 100% of the controls. Glycolytic and mitochondrial stress experimental data were expressed as the mean ± SEM and other data were expressed as the mean ± SD. Statistical analyses were performed using Prism 9 (GraphPad Software). Sample sizes (*n*) for each statistical analysis are provided in the relevant figure legends. *p* values of < 0.05 were considered statistically significant. No samples or animals were excluded from the analysis. Animals were randomly assigned to groups, and studies were not conducted blindly.

## Conflict of Interest

The authors declare no conflicts of interest.

## Author Contributions

Q.W., L.J.L., and X.Y.G contributed equally to this work. F.M., X.S., and Y.D.W. conceived of the study and initiated, designed, and supervised the experiments. Q.W., F.M., X.S., and Y.D.W. wrote the manuscript. Q.W., L.J.L., C.X., C.X.Z, L.Y.S., and J.L. performed experiments.

## Supporting information

Supporting Information

## Data Availability

The data that support the findings of this study are available from the corresponding author upon reasonable request.
