# Supplementary material for: Targeting GRP75 with a Chlorpromazine Derivative Inhibits Endometrial Cancer Progression Through GRP75–IP3R‐Ca2+‐AMPK Axis
Source: Adv Sci (Weinh). 2024 Feb 11;11(15):2304203. doi: 10.1002/advs.202304203 (PMC11022737; doi:10.1002/advs.202304203)
Supplement: Supplementary file 1 — Supporting Information [file ADVS-11-2304203-s001.pdf]

ADVANCED SCIENCE
Open Access

Supporting Information

for *Adv. Sci.*, DOI 10.1002/advs.202304203

Targeting GRP75 with a Chlorpromazine Derivative Inhibits Endometrial Cancer Progression Through GRP75–IP3R-Ca^{2+}-AMPK Axis

Qi Wang, Lijuan Li, Xiaoyan Gao, Chunxue Zhang, Chen Xu, Lingyi Song, Jian Li, Xiao Sun, Fei Mao* and Yudong Wang**

Supporting Information

Targeting GRP75 with a Chlorpromazine Derivative Inhibits Endometrial Cancer Progression through GRP75–IP3R-Ca^{2+}-AMPK axis

*Qi Wang, Lijuan Li, Xiaoyan Gao, Chunxue Zhang, Chen Xu, Lingyi Song, Jian Li, Xiao Sun *, Fei Mao *, Yudong Wang **

Supplementary Experimental Methods

Plasma protein binding using equilibrium dialysis: A 96-well equilibrium dialysis device and HTD 96 a/b regenerated cellulose membrane strips with a molecular mass cutoff of 12–14 kDa were obtained from HTDialysis LLC (Gales Ferry, CT). The dialysis device was assembled following the manufacturer's instructions.

Bioinformatics Analysis: The Kaplan–Meier Plotter (https://kmplot.com), GEPIA (http://gepia.cancer-pku.cn/), and Human Protein Atlas (http://www.proteinatlas.org/) were all utilized to analyze GRP75 expression in the EC microarray tissues and to assess overall survival and the relationship of GRP75 expression with prognosis.

Measurements of caspase 3/7 activities: A Caspase 3/7 Activity Assay Kit (Elabscience, China) was used to determine the caspase 3/7 activity. In brief, EC cells were seeded into 6 cm dishes and treated with JX57 (10 μmol/L) for 48 h. The activity of the caspase 3/7 was then determined according to the manufacturer's instructions.

RNA sequencing (RNA-seq) and enrichment analysis: Ishikawa cells were each divided into two subgroups, shNC and shHSPA9, with three replicate dishes for each cell type in each subgroup. TRIzol lysis buffer (Invitrogen, 15596026, Thermo Fisher Scientific, Waltham, MA, USA) was then added to the cell sample tube (1 mL TRIzol for 1×10^6 cells). The Illumina TruSeqTM RNA Sample Prep Kit (Illumina, San Diego, CA, USA) was used for library construction, and the Illumina NovaSeq 6000 sequencing platform was used for the sequencing experiments, both following the manufacturer's instructions. Gene expression levels were then calculated using RSEM (v1.3.1), and differential expression analysis was conducted using DESeq2 (v1.4.5) with a Q value ≤ 0.05. Subsequent analysis and data mining were performed using the Dr. Tom Multi-omics Data mining system (https://biosys.bgi.com).

Biological processes and enrichment analysis: Gene set enrichment analysis and assessment of the biological processes associated with differentially expressed genes in response to JX57 were performed using the WebGestalt.P[1] (https://www.webgestalt.org).

Supplementary Figures

Figure S1

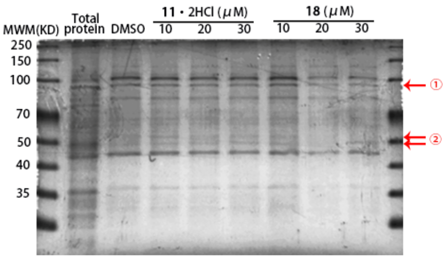

Figure S1. A Pierce™ Kinase Enrichment Kit with an ATP Probe was used to identify JX57 (11·2HCl) and JX66 (18) targets. The results for ISK cells are shown. The protein bands marked with red arrows were cut from the 10% SDS-PAGE gel and sent to Lumingbio for mass spectrometry analysis to determine their protein composition.

Figure S2

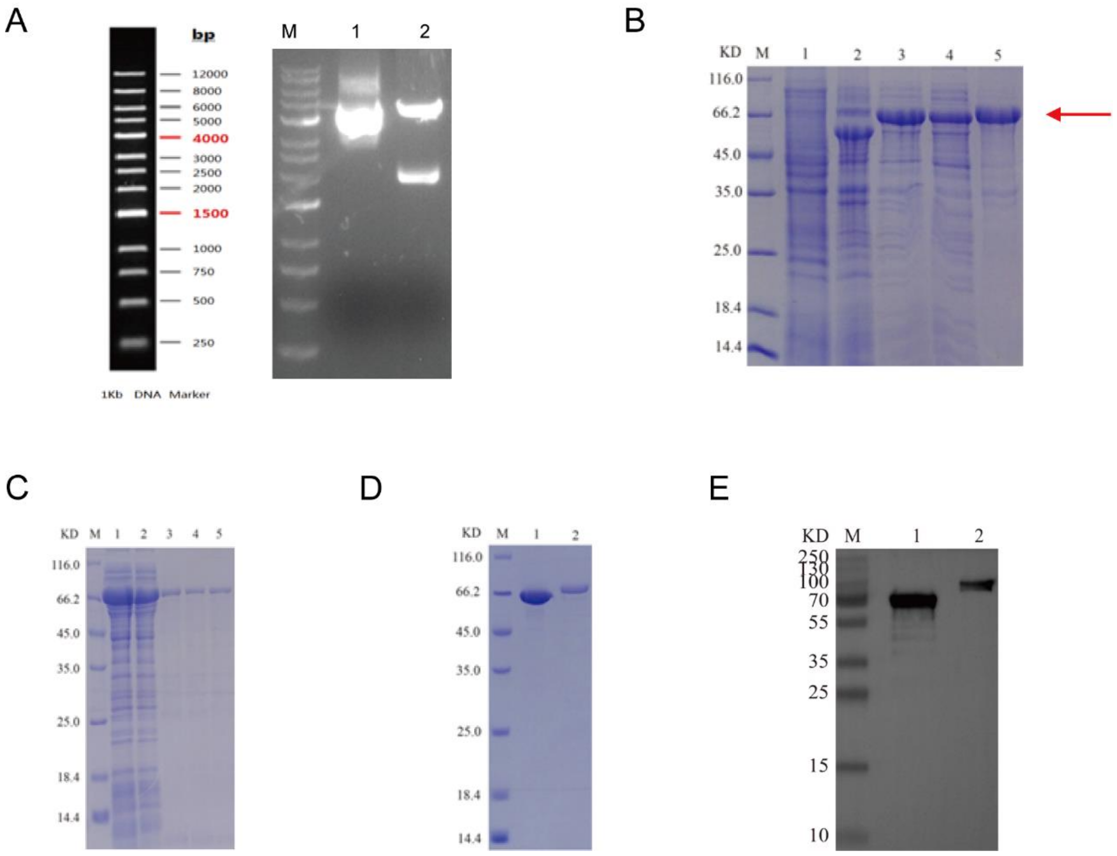

Figure S2. GRP75 recombinant protein synthesis. A) Restriction digestion map. Lane 1： Plasmid; Lane 2: Plasmid digested with NdeI-XbaI; Lane M: DNA marker. B)

Protein expression identification using SDS-PAGE analysis. Lane M: protein molecular quality standard; Lane 1: pCZN1 induction (no-load); Lane 2: not induced; Lane 3: after induction; Lane 4: induction of supernatant after crushing; Lane 5: induction of precipitation after crushing. C) Protein purification SDS-PAGE analysis. Lane M: protein molecular quality standard; Lane 1: processed samples after crushing; Lane 2: outflow; Lane 3–5: elution. D) Protein assay analysis. Lane M: protein molecular quality standard; Lane 1: 0.5 mg/mL BSA; Lane 2: purified sample. E) Protein identification using Western blotting identification analysis. Lane M: protein molecular quality standard; Lane 1: purified sample; Lane 2: multi-tag for positive control.

Figure S3

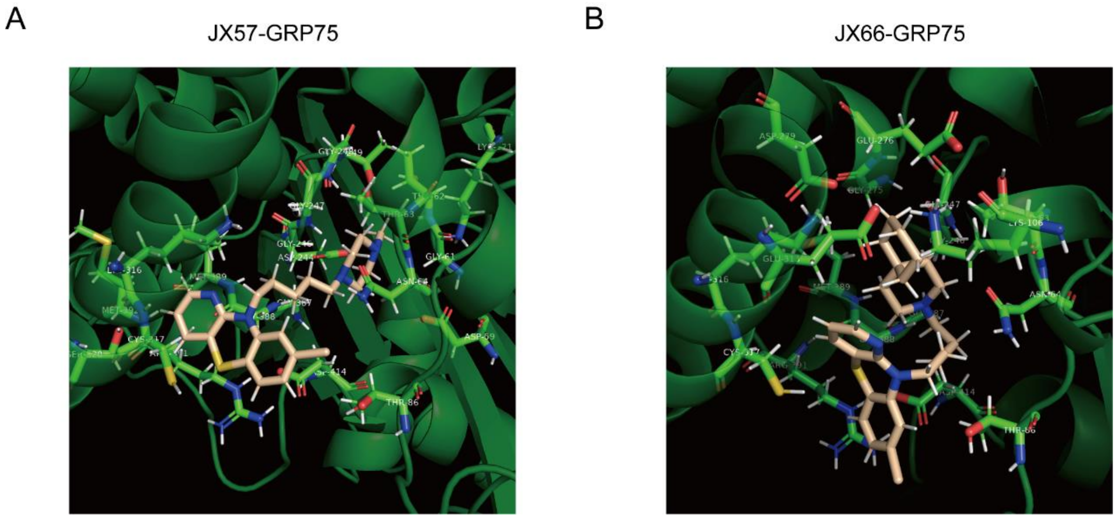

Figure S3. Molecular docking showed binding sites of JX57 and JX66 to GRP75. Binding sites of GRP75 to A) JX57 and B) JX66 were determined using Autodock.

Figure S4

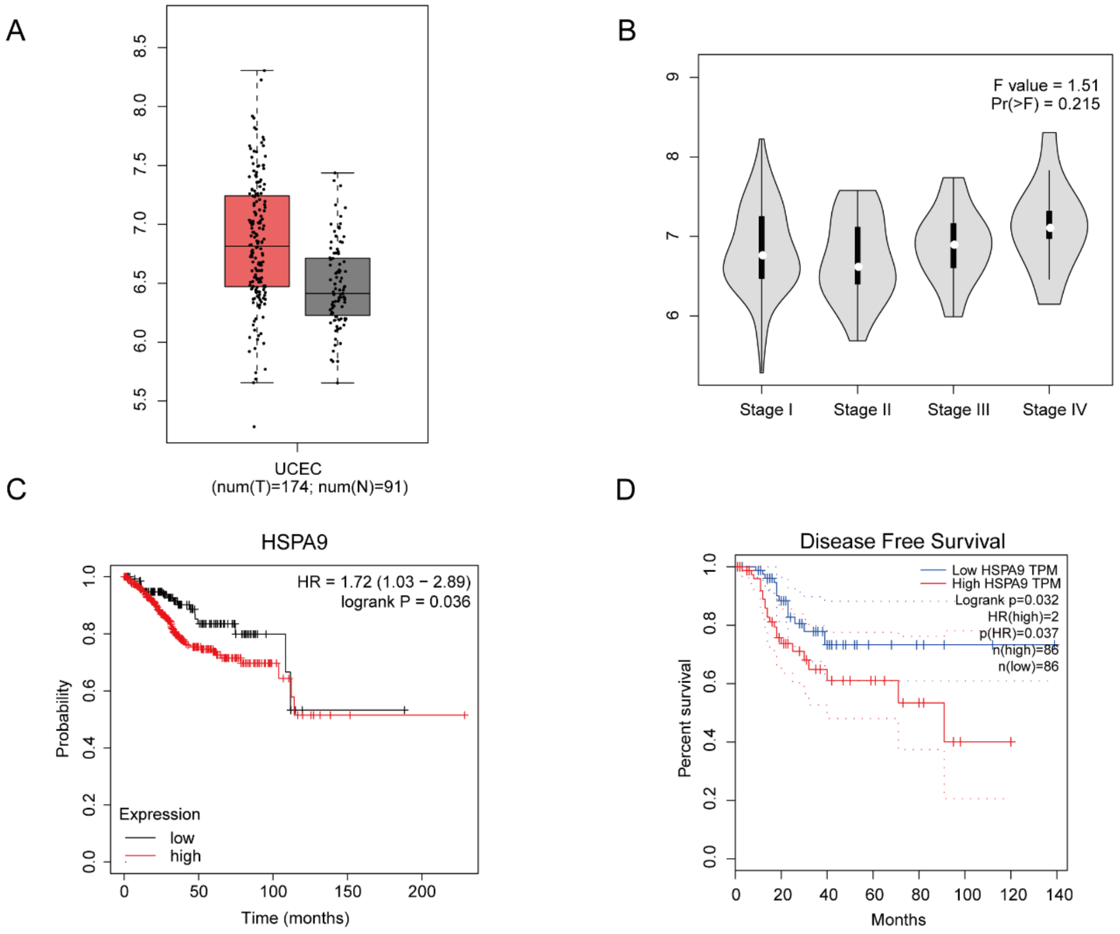

Figure S4. GRP75 was associated with a poor EC prognosis. A) Human Protein Atlas analysis revealed that GRP75 exhibits a higher expression level in EC tissues than in control tissues. B) The expression level of GRP75 was positively correlated with the stage of EC in the TCGA database. C) High expression of GRP75 was found to be associated with poor prognosis in EC using Kaplan–Meier Plotter analysis. D) A significant negative correlation was found between high GRP75 expression and disease-free survival in EC using GEPIA analysis.

Figure S5

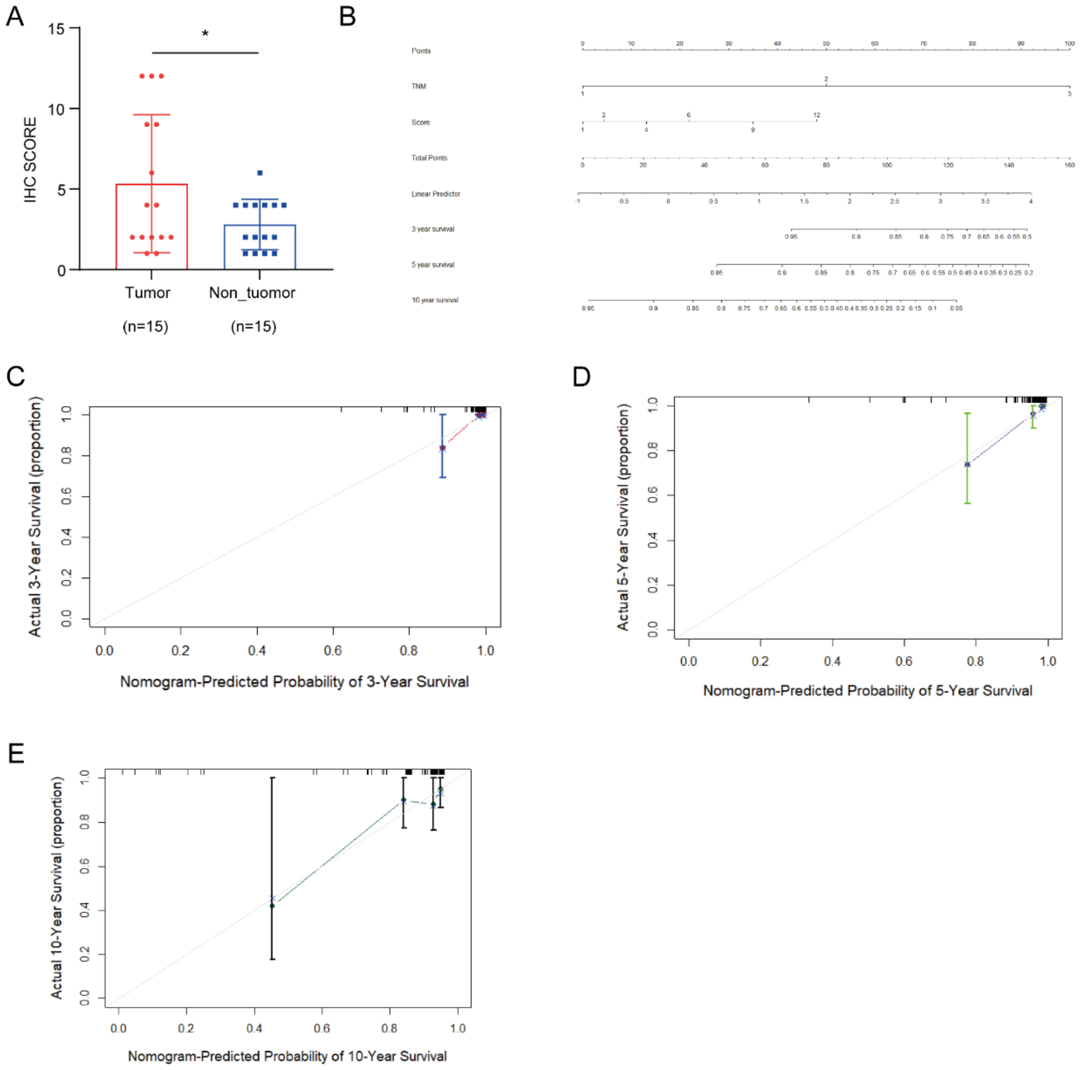

Figure S5. The nomogram survival prediction model predicts the overall survival of patients with EC. A) GRP75 expression was higher in EC tissues than in paracancerous tissues (n = 15). B–E) Each factor in the nomogram was assigned a weighted score, and the sum of the scores for each patient was used to predict the patient's 3-, 5-, and 10-year overall survival. Data show the mean ± SD; *P* values were calculated using unpaired *t*-tests; * $P < 0.05$.

Figure S6

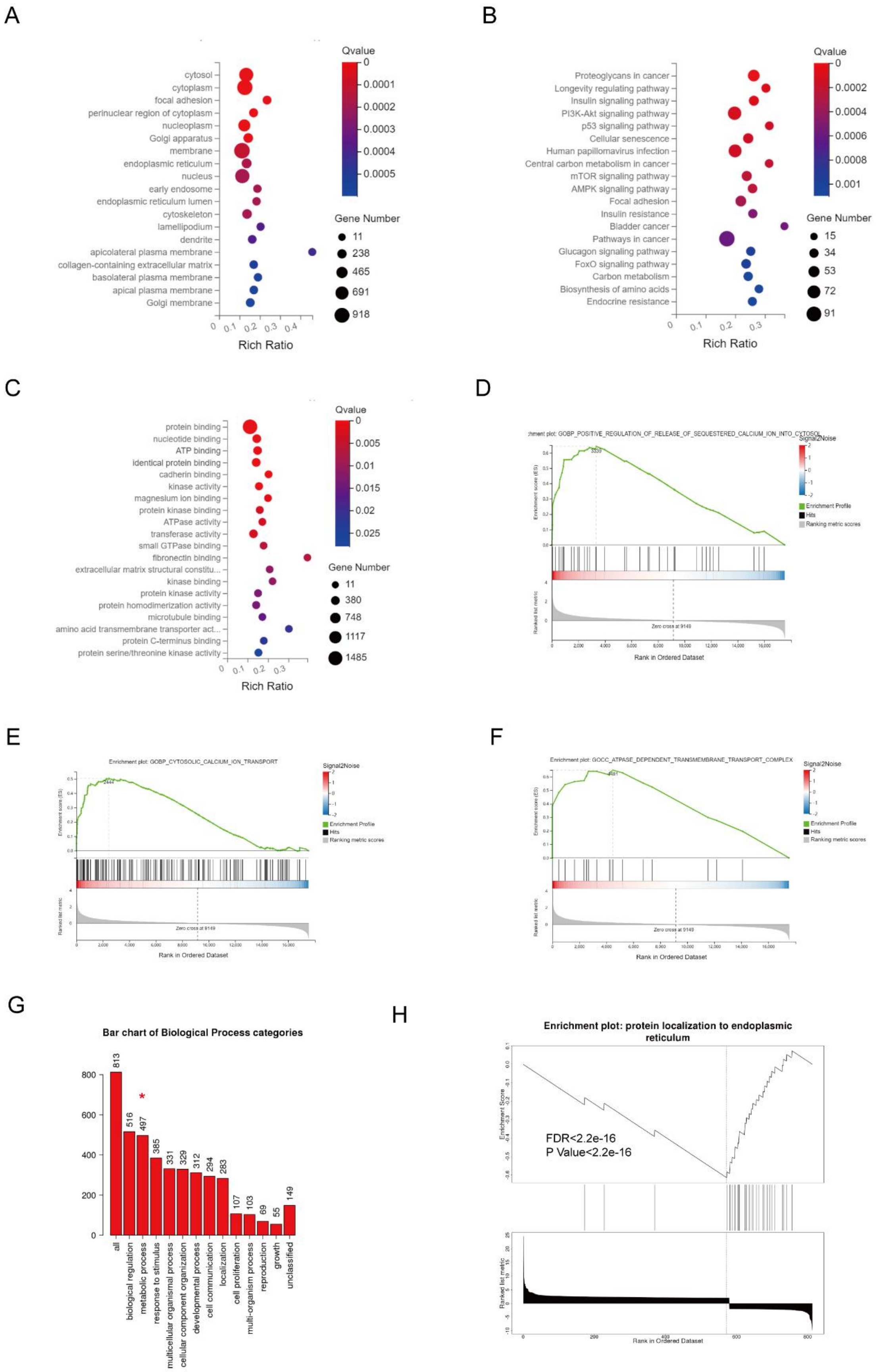

A
Qvalue
0
0.0001
0.0002
0.0003
0.0004
0.0005
cytosol
cytoplasm
focal adhesion
perinuclear region of cytoplasm
nucleoplasm
Golgi apparatus
membrane
endoplasmic reticulum
nucleus
early endosome
endoplasmic reticulum lumen
cytoskeleton
lamellipodium
dendrite
apicolateral plasma membrane
collagen-containing extracellular matrix
basolateral plasma membrane
apical plasma membrane
Golgi membrane
Gene Number
11
238
465
691
918
0
0.1
0.2
0.3
0.4
Rich Ratio
B
Qvalue
0
0.0002
0.0004
0.0006
0.0008
0.001
Proteoglycans in cancer
Longevity regulating pathway
Insulin signaling pathway
PI3K-Akt signaling pathway
p53 signaling pathway
Cellular senescence
Human papillomavirus infection
Central carbon metabolism in cancer
mTOR signaling pathway
AMPK signaling pathway
Focal adhesion
Insulin resistance
Bladder cancer
Pathways in cancer
Glucagon signaling pathway
FoxO signaling pathway
Carbon metabolism
Biosynthesis of amino acids
Endocrine resistance
Gene Number
15
34
53
72
91
0
0.1
0.2
0.3
Rich Ratio
C
Qvalue
0
0.005
0.01
0.015
0.02
0.025
protein binding
nucleotide binding
ATP binding
identical protein binding
cadherin binding
kinase activity
magnesium ion binding
protein kinase binding
ATPase activity
transferase activity
small GTPase binding
fibronectin binding
extracellular matrix structural constitu...
kinase binding
protein kinase activity
protein homodimerization activity
microtubule binding
amino acid transmembrane transporter act...
protein C-terminus binding
protein serine/threonine kinase activity
Gene Number
11
380
748
1117
1485
0
0.1
0.2
0.3
Rich Ratio
D
Enrichment plot: GOBP_POSITIVE_REGULATION_OF_RELEASE_OF_SEQUESTERED_CALCIUM_ION_INTO_CYTOSOL
Signal2Noise
Enrichment Profile
Hits
Ranking metric scores
Zero cross at 9149
Rank in Ordered Dataset
E
Enrichment plot: GOBP_CYTOSOLIC_CALCIUM_ION_TRANSPORT
Signal2Noise
Enrichment Profile
Hits
Ranking metric scores
Zero cross at 9149
Rank in Ordered Dataset
F
Enrichment plot: GOCC_ATPASE_DEPENDENT_TRANSMEMBRANE_TRANSPORT_COMPLEX
Signal2Noise
Enrichment Profile
Hits
Ranking metric scores
Zero cross at 9149
Rank in Ordered Dataset
G
Bar chart of Biological Process categories
813
516
497
385
331
329
312
294
283
107
103
69
55
149
800
600
400
200
0
*
all
biological regulation
metabolic process
response to stimulus
multicellular organismal process
cellular component organization
developmental process
cell communication
localization
cell proliferation
multi-organism process
reproduction
growth
unclassified
H
Enrichment plot: protein localization to endoplasmic reticulum
Enrichment Score
FDR<2.2e-16
P Value<2.2e-16
Ranked list metric
Rank in Ordered Dataset

Figure S6. Transcriptome sequencing analysis. A) GO cellular component enrichment analysis indicated that differentially expressed genes upon GRP75 knockdown were significantly enriched in the ER and cytoplasm. B) KEGG pathway enrichment analysis indicated significant enrichment in the AMPK pathway for differential gene alterations caused by GRP75 knockdown. C) GO molecular function enrichment analysis indicated that differential gene alterations caused by GRP75 knockdown were significantly enriched in ATP-binding and ATPase activity. D–F) GSEA indicated that differentially expressed genes upon GRP75 knockdown were significantly enriched in calcium ion-related transport pathways as well as transport membrane complexes. G) Functional analysis of the transcriptome sequencing for the JX57-treated EC cells. The results indicate high levels of enrichment for the metabolic processes in the biological process category. H) GSEA analysis of the JX57 treated EC cells. The results indicate that the process of protein localization to the endoplasmic reticulum is down-regulated.

Figure S7

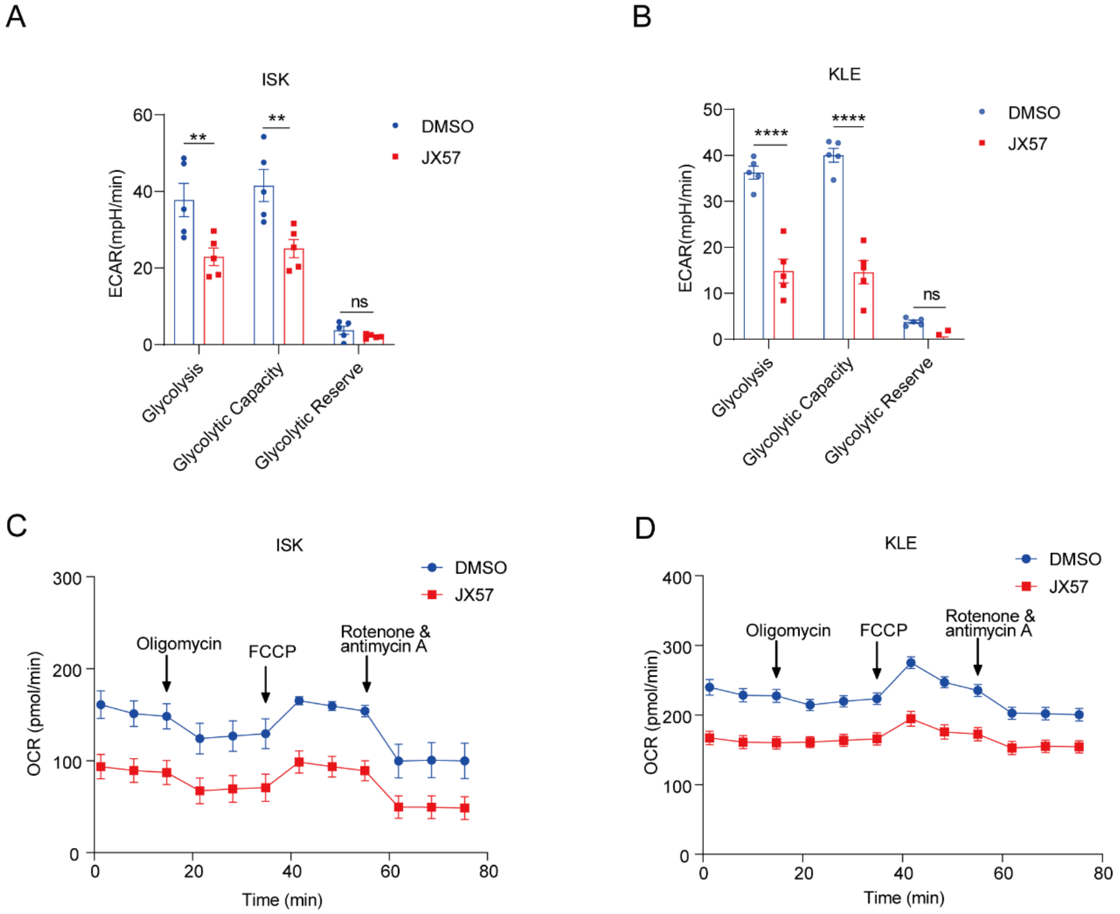

Figure S7. JX57 inhibited glycolysis and oxidative phosphorylation in EC cells. A–B) JX57 significantly inhibits the glycolytic and glycolytic capacity of the EC cells (n = 6). C–D) The effects of JX57 on oxidative phosphorylation in EC cells were determined using an oxygen consumption assay (n = 6). Data show the mean ± SEM; P values were calculated using a two-way ANOVA; * $P < 0.05$, ** $P < 0.01$, *** $P < 0.001$, and ****

$P < 0.0001$.

Figure S8

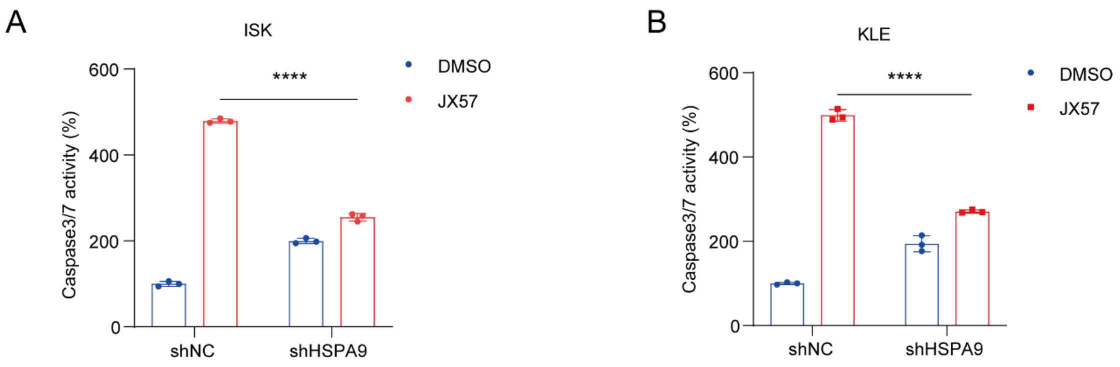

Figure S8. JX57 triggered EC cell apoptosis via GRP75. A) GRP75-deficient ISK and B) KLE cells were treated with JX57 (10 μmol/L) for 48 h, and a Caspase 3/7 activity assay was then used for detection (n = 3). Data show the mean ± SD; P values were calculated using two-way ANOVA; **** $P < 0.0001$.

Supplementary Tables

Table S1. Plasma protein binding rates for JX57 and JX66.

Compd.	%Unbound [a]	% Bound
CPZ	1.56	98.44
JX57	0.84	99.16
JX66	-	-

[a] The % Unbound/% Bound values were not available because no detectable peaks were seen in free samples.

Table S2. Potential target proteins for JX57 identified by ABPP and mass spectrometry.

UniProt	**Protein name**	**Gene name**	**Description**
**P38646**	GRP75: Stress-70 protein, mitochondrial	*HSPA9*	The encoded protein is primarily localized to the mitochondria but is also found in the ER. This protein plays a role in cell proliferation, stress response, and maintenance of the mitochondria.
**P13804**	ETFA: Electron transfer flavoprotein subunit alpha	*ETFA*	Heterodimeric electron transfer flavoprotein that accepts electrons from several mitochondrial dehydrogenases
**Q01813**	PFKAP: Protein ATP-dependent 6-phosphofructokinase	*PFKP*	Catalyzes the phosphorylation of D-fructose 6-phosphate to fructose 1,6-bisphosphate by ATP.
**Q14643**	ITPR1: Inositol 1,4,5-trisphosphate receptor type 1	*ITPR1*	Plays a role in ER stress-induced apoptosis.
**P11498**	PYC: Pyruvate carboxylase	*PC*	Pyruvate carboxylase catalyzes a two-step reaction, involving the ATP-dependent carboxylation of the covalently attached biotin in the first step.
**P54886**	P5CS: Delta-1-pyrroline-5-carboxylate synthase	*ALDH18A1*	Bifunctional enzyme that converts glutamate to glutamate 5-semialdehyde.
**Q71U36**	TBA1A: Tubulin alpha-1A chain	*TUBA1A*	Tubulin is the major constituent of microtubules.
**Q16891**	MIC60: MICOS complex subunit MIC60	*IMMT*	Component of the MICOS complex, a large protein complex of the mitochondrial inner membrane.

Table S3. Ingenuity canonical pathways of TOP6

Ingenuity Canonical Pathways	-log(p-value)	Ratio	z-score
EIF2 signaling	1.48E01	1.75E−01	−2.294
mTOR signaling	6.91E00	1.22E−01	1.732
Huntington's disease signaling	5.93E00	1.01E−01	2.673
Prolactin signaling	5.79E00	1.58E−01	2.111
Regulation of eIF4 and p70S6K signaling	5.75E00	1.19E−01	1.890
Insulin receptor signaling	5.67E00	1.32E−01	1.213

Table S4. Correlation between GRP75 expression and clinicopathological parameters in EMC1351.

Clinical parameters		Case	GRP75 expression		χ2	*P* value
			Low (*n*)	High (*n*)		
Age (years)	＜ 55	49	24	25	8.142	0.004*
	≥ 55	61	14	47		
Tumor size (cm)	＜ 5	59	18	41	1.470	0.225
	≥ 5	35	15	20		
Pathologic_T	T1	93	31	62	0.391	0.532
	T2/T3	17	7	10		
Pathologic_N	N0	105	36	69	0.000	1.000
	N1	5	2	3		
TNM stage	I	91	31	60	0.054	0.817
	II-III-IV	19	7	12		

Histological grade	High differentiation	33	16	17	9.826	0.043*
	Moderately high differentiation	23	8	15		
	Moderate differentiation	31	5	26		
	Moderately low differentiation	14	8	6		
	Low differentiation	7	1	6		
Overall survival status	Alive	96	37	59	4.029	0.045*
	Dead	14	1	13		

Table S5. Results of univariate and multivariate analysis for overall survival in EMC1351

**Variable**	**Univariate analysis**		**Multivariate analysis**	
	***P* value**	**HR (95% CI)**	**P-value**	**HR (95% CI)**
Age（< 55 vs ≥ 55 years）	0.930	1.049（0.363–3.025）		
Pathologic_T（T1 vs T2/T3）	0.001	5.730（1.951–16.833）	0.188	0.157（0.010–2.463）
Pathologic_N（N0 vs N1）	0.000	11.433（2.993–43.675）	0.728	1.507（0.150–15.148）
TNM（I vs II/III/IV）	0.000	10.075（3.407–29.800）	0.009*	53.083（2.646–1064.811）
Tumor_size（< 5 vs ≥ 5 cm）	0.307	1.805（0.581–5.606）		
Differentiation（High differentiation/ Moderately high differentiation/ Moderate differentiation vs Moderately low differentiation/ Low differentiation）	0.103-	2.484（0.832–7.420）	0.670	1.312（0.376–4.577）
GRP75 Expression（low vs high）	0.054-	7.395（0.967–56.549）	0.042*	8.622（1.085–68.543）
P53（positive vs negative）	0.607	1.397（0.391–4.993）		
ER（positive vs negative）	0.687	1.375（0.292–6.480）		
PR（positive vs negative）	0.913	1.089（0.235–5.053）		
KI_67（positive vs negative）	0.872	1.141（0.229–5.675）		

To avoid missing important factors, the *P* value was relaxed to 0.15, so that the T, N, TNM, differentiation, and GRP75 expression were all included in the multivariate analysis.

Reference:

[1] LIAO Y, WANG J, JAEHNIG E J, et al. WebGestalt 2019: gene set analysis toolkit with revamped UIs and APIs [J]. Nucleic Acids Res, 2019, 47(W1): W199-W205.
